# *N*-[(*E*)-Quinolin-2-yl­methyl­idene]-1,2,4-triazol-4-amine hemihydrate

**DOI:** 10.1107/S2414314620001340

**Published:** 2020-02-03

**Authors:** Nurulizzatul Ningsheh M. Shahri, Nur Halilatul Sadiqin Omar Ali, Malai Haniti Sheikh Abdul Hamid, Aminul Huq Mirza, Anwar Usman, Md Rejaul Hoq, Mohammad R. Karim

**Affiliations:** a Chemical Sciences, Faculty of Science, Universiti Brunei Darussalam, Jalan Tungku Link BE1410, Negara Brunei Darussalam; b Department of Chemistry, Tennessee State University, 2500 John A. Merritt Blvd., Nashville, Tennessee, TN 37209, USA; Okayama University, Japan

**Keywords:** crystal structure, Schiff base, green synthesis, triazole, inter­molecular hydrogen bond

## Abstract

The title hemihydrate, C_12_H_9_N_5_·0.5H_2_O, was isolated from the condensation reaction of quinoline-2-carbaldehyde with 4-amino-4*H*-1,2,4-triazole. The Schiff base mol­ecule adopts an *E* configuration about the C=N bond and is approximately planar, with a dihedral angle between the quinoline ring system and the 1,2,4-triazole ring of 12.2 (1)°. In the crystal, one water mol­ecule bridges two Schiff base mol­ecules *via* O—H⋯N hydrogen bonds. The Schiff base mol­ecules are inter­connected by π–π stacking inter­actions [centroid-centroid distances of 3.7486 (7) and 3.9003 (7) Å] into columns along [1  0].

## Abstract 2

In the title hemihydrate, the Schiff base mol­ecule adopts an *E* configuration about the C=N bond and is approximately planar, with a dihedral angle between the quinoline ring system and the 1,2,4-triazole ring of 12.2 (1)°. In the crystal, one water mol­ecule bridges two Schiff base mol­ecules *via* O—H⋯N hydrogen bonds.

## Graphical abstract

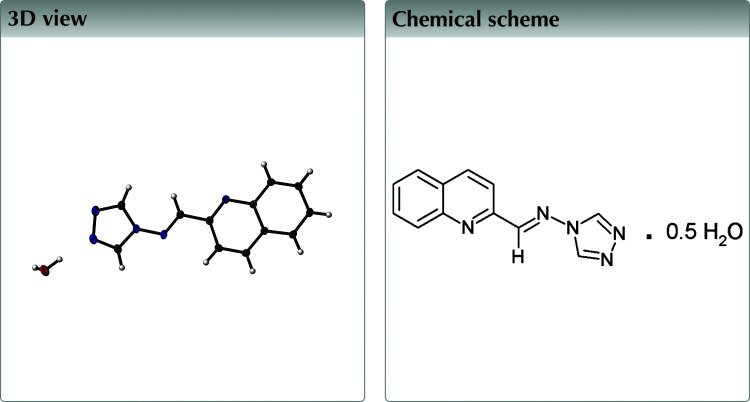

## Structure description

Schiff bases containing a heterocyclic 1,2,4-triazole moiety have been investigated for their bioactivities and pharmaceutical applications (Bhalgat *et al.*, 2014 ▸; Saadaoui *et al.*, 2019 ▸; Zhang *et al.*, 2019 ▸; Akin *et al.*, 2019 ▸). Recently, the structures of Schiff bases obtained from 3-amino-1*H*-1,2,4-triazole have been reported in detail (Kołodziej *et al.*, 2019 ▸). In the present work, we report the crystal structure of a new Schiff base, namely *N*-[(*E*)-quinolin-2-yl­methyl­idene]-1,2,4-triazol-4-amine hemihydrate.

**Figure 1:**
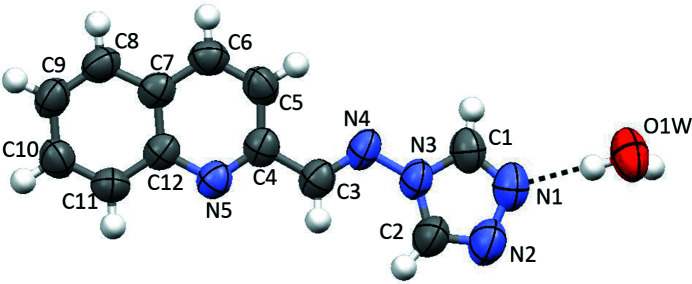
The mol­ecular structure of the title compound, showing the atom-labelling scheme and 50% probability displacement ellipsoids.

Fig. 1 ▸ illustrates the mol­ecular structure of the title compound with the atomic numbering. The bond lengths and angles are within the expected range and normal values. In particular, C3—C4, C3—N4 and N3—N4 bond lengths are 1.475 (2), 1.279 (2) and 1.387 (2) Å, respectively, confirming its Schiff base structure. The title compound as a whole is a conjugated system with two aromatic fragments (quinoline and triazole) linked by the azomethine C3=N4 double bond and is approximately planar, adopting an *E* configuration. The azomethine (N4/C3/H3) fragment is twisted by 7.36 (9)° with respect to the quinoline ring system, and the dihedral angle between the quinoline ring system and the 1,2,4-triazole ring is 12.2 (1)°.

**Figure 2:**
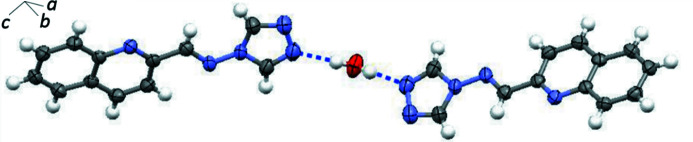
A partial packing diagram of the title compound, showing two Schiff base mol­ecules linked by two identical inter­molecular O—H⋯N hydrogen bonds.

**Figure 3:**
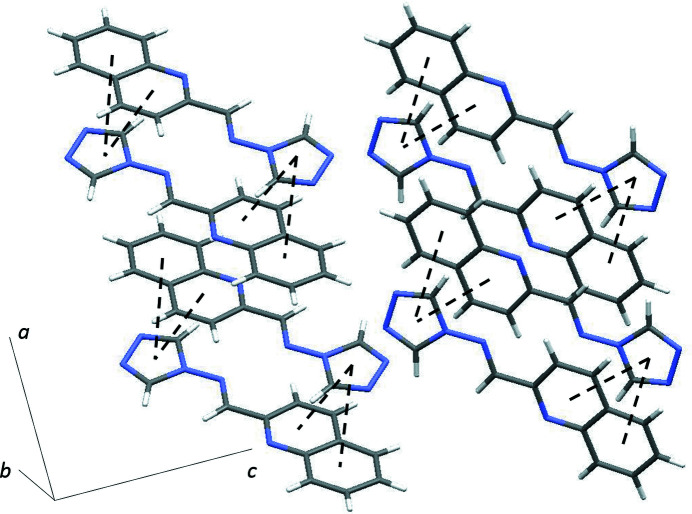
A partial packing diagram of the title compound, showing a mol­ecular column formed by π–π stacking inter­actions.

In the crystal, the O atom of water mol­ecule lies on a twofold rotation axis and also close to the plane of the adjacent quinoline ring system, deviating by 0.157 Å. As a result, the water mol­ecule forms a symmetric system of O—H⋯N hydrogen bonds (Table 1 ▸) with two Schiff base mol­ecules (Fig. 2 ▸); the hydrogen bonds link the water mol­ecule with the 1,2,4-triazole rings. The Schiff base mol­ecules are stacked, forming mol­ecular columns along [1 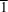 0] (Fig. 3 ▸) by π–π stacking inter­actions with centroid–centroid distances of 3.7486 (7) Å between the C1/N1/N2/C2/N3 and C7–C12 rings, and 3.9003 (7) Å between the C1/N1/N2/C2/N3 and N5/C4–C7/C12 rings.

## Synthesis and crystallization

A solution of quinoline-2-carbaldehyde (1.00 g, 6.0 mmol) was mixed with an equimolar solution of 4-amino-4*H*-1,2,4-triazole (0.54 g, 6.0 mmol) in a mixture of absolute ethanol and chloro­form (1:1) (20 ml). Glacial acetic acid (2 drops) was added into the reaction mixture, followed by heating at 351 K for 6 h for complete conversion to the product (as confirmed by TLC analysis). The mixture was then kept in an ambient environment for two weeks. The crude product obtained was recrystallized from ethyl acetate solution, giving clear brown crystals (yield 55%) suitable for X-ray analysis. Presumably the water molecule of crystallization was absorbed from the atmosphere or as a by product during the synthesis. Analysis: C_12_H_9_N_5_ (%): C 64.56, H 4.06, N 31.37; found (%): C 64.74, H 4.34, N 30.67; ^1^H NMR (DMSO-*d*_6_): *δ* 9.35 (*s*, 2H, H-1,2), 9.27 (*s*, 1H, CH=N, H-3), 8.56 (*d*, 1H, H-5. *J* = 8.12 Hz 1H), 8.17 (*d*, 1H, H-6. *J* = 8.12 Hz), 8.12 (*m*, 2H, H-8,11), 7.89 (*t*, 1H, H-10, *J* = 6.8 Hz), 7.75 (*t*, 1H, H-9, *J* = 6.8 Hz). ^13^C NMR (DMSO-*d*_6_): *δ* 157.55 (CH=N, C3), 152.17 (C1,2), 147.83, 139.79, 137.98, 131.12, 129.63, 128.92, 128.68, 118.36. IR (KBr, cm^−1^): 3103 (Aryl C—H), 1597 (C=N), 1051 (N—N), 957 (C=S). EI—MS calculated for C_12_H_9_N_5_ [*M*]^+^: 223.24, Found: 223. m.p. 487–489 K.

The title compound was also synthesized using a green synthesis method. A solution of 4-amino-4*H*-1,2,4-triazole (0.11 g, 1.3 mmol) in 5 ml of distilled water was added to quinoline-2-carbaldehyde (0.20 g, 1.3 mmol) in 5 ml of distilled water. The resulting mixture was then stirred at room temperature for 1 h while the reaction progress was monitored by TLC. The clear light-brown crude product formed qu­anti­tatively after 1 h and was vacuum filtered, dried and recrystallized from ethyl acetate solution to give clear dark-brown crystals with 60% yield. The recrystallized compound from the conventional synthesis method and that from the green method were confirmed to be identical as given by the similar data from FTIR, TLC, MS, and melting-point measurements.

## Refinement

Crystal data, data collection and structure refinement details of the title compound are summarized in Table 2 ▸.

## Supplementary Material

Crystal structure: contains datablock(s) I. DOI: 10.1107/S2414314620001340/is4041sup1.cif

Structure factors: contains datablock(s) I. DOI: 10.1107/S2414314620001340/is4041Isup2.hkl

Supporting information file. DOI: 10.1107/S2414314620001340/is4041Isup3.cml

CCDC reference: 1961612

Additional supporting information:  crystallographic information; 3D view; checkCIF report

## Figures and Tables

**Table 1:** Hydrogen-bond geometry (Å, °)

*D*—H⋯*A*	*D*—H	H⋯*A*	*D*⋯*A*	*D*—H⋯*A*
O1*W*—H1*W*⋯N1	0.914 (18)	1.939 (18)	2.8422 (11)	169.3 (17)

**Table 2:** Experimental details

Crystal data	
Chemical formula	C_12_H_9_N_5_·0.5H_2_O
*M*_r_	232.25
Crystal system, space group	Monoclinic, *C*2/*c*
Temperature (K)	100
*a*, *b*, *c* (Å)	13.7212 (12), 7.5047 (6), 21.1686 (18)
β (°)	100.524 (2)
*V* (Å^3^)	2143.1 (3)
*Z*	8
Radiation type	Cu *K*α
μ (mm^−1^)	0.79
Crystal size (mm)	0.36 × 0.26 × 0.17

Data collection	
Diffractometer	Bruker D8 Venture
Absorption correction	Multi-scan (*SADABS*; Bruker, 2014 ▸)
*T*_min_, *T*_max_	0.626, 0.754
No. of measured, independent and observed [*I* > 2σ(*I*)] reflections	36175, 2200, 2142
*R*_int_	0.036
(sin θ/λ)_max_ (Å^−1^)	0.625

Refinement	
*R*[*F* ^2^ > 2σ(*F* ^2^)], *wR*(*F* ^2^), *S*	0.036, 0.090, 1.09
No. of reflections	2200
No. of parameters	164
H-atom treatment	H atoms treated by a mixture of independent and constrained refinement
Δρ_max_, Δρ_min_ (e Å^−3^)	0.22, −0.23

Computer programs: *APEX3* (Bruker, 2014 ▸), *SAINT* (Bruker, 2014 ▸), *SHELXT* (Sheldrick, 2015*a* ▸), *SHELXL2014* (Sheldrick, 2015*b* ▸), *SHELXTL* (Sheldrick, 2008 ▸).

## full crystallographic data

### Crystal data

**Table:**

C_12_H_9_N_5_·0.5H_2_O	*F*(000) = 968
*M**_r_* = 232.25	*D*_x_ = 1.440 Mg m^−^^3^
Monoclinic, *C*2/*c*	Cu *K*α radiation, λ = 1.54178 Å
*a* = 13.7212 (12) Å	Cell parameters from 9219 reflections
*b* = 7.5047 (6) Å	θ = 4.3–74.4°
*c* = 21.1686 (18) Å	µ = 0.79 mm^−^^1^
β = 100.524 (2)°	*T* = 100 K
*V* = 2143.1 (3) Å^3^	Block, brown
*Z* = 8	0.36 × 0.26 × 0.17 mm

### Data collection

**Table:**

Bruker D8 Venture diffractometer	2142 reflections with *I* > 2σ(*I*)
Radiation source: Sealed Microfocus Source	*R*_int_ = 0.036
φ and ω scans	θ_max_ = 74.5°, θ_min_ = 4.3°
Absorption correction: multi-scan (SADABS; Bruker, 2014)	*h* = −17→17
*T*_min_ = 0.626, *T*_max_ = 0.754	*k* = −9→9
36175 measured reflections	*l* = −25→26
2200 independent reflections	

### Refinement

**Table:**

Refinement on *F*^2^	Hydrogen site location: mixed
Least-squares matrix: full	H atoms treated by a mixture of independent and constrained refinement
*R*[*F*^2^ > 2σ(*F*^2^)] = 0.036	*w* = 1/[σ^2^(*F*_o_^2^) + (0.0426*P*)^2^ + 1.6998*P*] where *P* = (*F*_o_^2^ + 2*F*_c_^2^)/3
*wR*(*F*^2^) = 0.090	(Δ/σ)_max_ < 0.001
*S* = 1.09	Δρ_max_ = 0.22 e Å^−^^3^
2200 reflections	Δρ_min_ = −0.22 e Å^−^^3^
164 parameters	Extinction correction: SHELXL2014 (Sheldrick, 2015*b*), Fc^*^=kFc[1+0.001xFc^2^λ^3^/sin(2θ)]^-1/4^
0 restraints	Extinction coefficient: 0.0104 (4)

### Special details

**Table:**

Geometry. All esds (except the esd in the dihedral angle between two l.s. planes) are estimated using the full covariance matrix. The cell esds are taken into account individually in the estimation of esds in distances, angles and torsion angles; correlations between esds in cell parameters are only used when they are defined by crystal symmetry. An approximate (isotropic) treatment of cell esds is used for estimating esds involving l.s. planes.

### Fractional atomic coordinates and isotropic or equivalent isotropic displacement parameters (Å^2^)

**Table:**

	*x*	*y*	*z*	*U*_iso_*/*U*_eq_	
N1	0.14712 (7)	0.46460 (13)	0.69779 (5)	0.0216 (2)	
N2	0.22716 (7)	0.56151 (14)	0.73141 (5)	0.0234 (2)	
N3	0.24350 (6)	0.54032 (12)	0.63061 (4)	0.0168 (2)	
N4	0.27388 (7)	0.54247 (12)	0.57163 (4)	0.0172 (2)	
N5	0.46063 (6)	0.73766 (12)	0.50056 (4)	0.0156 (2)	
C1	0.15910 (8)	0.45416 (15)	0.63833 (5)	0.0188 (2)	
H1	0.1152	0.3949	0.6050	0.023*	
C2	0.28338 (8)	0.60519 (16)	0.69030 (5)	0.0207 (3)	
H2	0.3430	0.6720	0.7003	0.025*	
C3	0.35238 (8)	0.62997 (14)	0.56723 (5)	0.0164 (2)	
H3	0.3880	0.6932	0.6030	0.020*	
C4	0.38588 (7)	0.62967 (14)	0.50486 (5)	0.0155 (2)	
C5	0.33874 (8)	0.51790 (15)	0.45428 (5)	0.0176 (2)	
H5	0.2856	0.4423	0.4602	0.021*	
C6	0.37095 (8)	0.52081 (14)	0.39711 (5)	0.0173 (2)	
H6	0.3398	0.4485	0.3624	0.021*	
C7	0.45134 (7)	0.63285 (14)	0.38995 (5)	0.0150 (2)	
C8	0.49101 (8)	0.63959 (14)	0.33287 (5)	0.0176 (2)	
H8	0.4652	0.5636	0.2979	0.021*	
C9	0.56649 (8)	0.75496 (15)	0.32765 (5)	0.0181 (2)	
H9	0.5923	0.7598	0.2890	0.022*	
C10	0.60599 (8)	0.86659 (15)	0.37961 (5)	0.0181 (2)	
H10	0.6571	0.9485	0.3751	0.022*	
C11	0.57180 (8)	0.85879 (14)	0.43664 (5)	0.0167 (2)	
H11	0.6008	0.9319	0.4717	0.020*	
C12	0.49343 (7)	0.74173 (13)	0.44309 (5)	0.0145 (2)	
O1W	0.0000	0.27774 (16)	0.7500	0.0257 (3)	
H1W	0.0437 (13)	0.351 (3)	0.7347 (9)	0.053 (5)*	

### Atomic displacement parameters (Å^2^)

**Table:**

	*U*^11^	*U*^22^	*U*^33^	*U*^12^	*U*^13^	*U*^23^
N1	0.0192 (5)	0.0245 (5)	0.0230 (5)	0.0016 (4)	0.0086 (4)	0.0027 (4)
N2	0.0225 (5)	0.0295 (5)	0.0201 (5)	0.0004 (4)	0.0087 (4)	0.0000 (4)
N3	0.0151 (4)	0.0211 (5)	0.0154 (4)	0.0012 (3)	0.0059 (3)	0.0007 (3)
N4	0.0163 (4)	0.0227 (5)	0.0138 (4)	0.0030 (3)	0.0056 (3)	0.0015 (3)
N5	0.0146 (4)	0.0177 (4)	0.0147 (4)	0.0019 (3)	0.0031 (3)	0.0003 (3)
C1	0.0155 (5)	0.0203 (5)	0.0218 (5)	0.0016 (4)	0.0062 (4)	0.0021 (4)
C2	0.0194 (5)	0.0269 (6)	0.0167 (5)	−0.0003 (4)	0.0060 (4)	−0.0016 (4)
C3	0.0153 (5)	0.0184 (5)	0.0155 (5)	0.0026 (4)	0.0033 (4)	0.0007 (4)
C4	0.0133 (5)	0.0181 (5)	0.0150 (5)	0.0035 (4)	0.0024 (4)	0.0022 (4)
C5	0.0136 (5)	0.0209 (5)	0.0178 (5)	−0.0017 (4)	0.0018 (4)	0.0024 (4)
C6	0.0164 (5)	0.0190 (5)	0.0153 (5)	−0.0005 (4)	−0.0001 (4)	−0.0002 (4)
C7	0.0144 (5)	0.0165 (5)	0.0135 (5)	0.0022 (4)	0.0008 (4)	0.0018 (4)
C8	0.0193 (5)	0.0205 (5)	0.0125 (5)	0.0000 (4)	0.0015 (4)	−0.0002 (4)
C9	0.0174 (5)	0.0241 (6)	0.0132 (5)	0.0009 (4)	0.0037 (4)	0.0022 (4)
C10	0.0149 (5)	0.0210 (6)	0.0182 (5)	−0.0021 (4)	0.0027 (4)	0.0021 (4)
C11	0.0160 (5)	0.0184 (5)	0.0153 (5)	−0.0010 (4)	0.0012 (4)	−0.0009 (4)
C12	0.0136 (5)	0.0164 (5)	0.0132 (5)	0.0032 (4)	0.0020 (4)	0.0010 (4)
O1W	0.0228 (6)	0.0227 (6)	0.0356 (7)	0.000	0.0153 (5)	0.000

### Geometric parameters (Å, º)

**Table:**

N1—C1	1.3011 (14)	C5—H5	0.9500
N1—N2	1.3985 (14)	C6—C7	1.4169 (15)
N2—C2	1.3057 (14)	C6—H6	0.9500
N3—C1	1.3621 (14)	C7—C8	1.4140 (14)
N3—C2	1.3714 (14)	C7—C12	1.4245 (14)
N3—N4	1.3866 (12)	C8—C9	1.3698 (15)
N4—C3	1.2793 (14)	C8—H8	0.9500
N5—C4	1.3233 (14)	C9—C10	1.4099 (15)
N5—C12	1.3724 (13)	C9—H9	0.9500
C1—H1	0.9500	C10—C11	1.3739 (14)
C2—H2	0.9500	C10—H10	0.9500
C3—C4	1.4752 (14)	C11—C12	1.4143 (14)
C3—H3	0.9500	C11—H11	0.9500
C4—C5	1.4196 (15)	O1W—H1W	0.914 (18)
C5—C6	1.3623 (15)		
			
C1—N1—N2	107.30 (9)	C5—C6—C7	119.32 (10)
C2—N2—N1	107.22 (9)	C5—C6—H6	120.3
C1—N3—C2	105.24 (9)	C7—C6—H6	120.3
C1—N3—N4	121.04 (9)	C8—C7—C6	122.77 (9)
C2—N3—N4	133.67 (9)	C8—C7—C12	119.37 (9)
C3—N4—N3	117.85 (9)	C6—C7—C12	117.86 (9)
C4—N5—C12	117.27 (9)	C9—C8—C7	120.42 (10)
N1—C1—N3	110.36 (10)	C9—C8—H8	119.8
N1—C1—H1	124.8	C7—C8—H8	119.8
N3—C1—H1	124.8	C8—C9—C10	120.08 (10)
N2—C2—N3	109.88 (10)	C8—C9—H9	120.0
N2—C2—H2	125.1	C10—C9—H9	120.0
N3—C2—H2	125.1	C11—C10—C9	121.06 (10)
N4—C3—C4	117.94 (9)	C11—C10—H10	119.5
N4—C3—H3	121.0	C9—C10—H10	119.5
C4—C3—H3	121.0	C10—C11—C12	119.93 (10)
N5—C4—C5	124.21 (10)	C10—C11—H11	120.0
N5—C4—C3	115.64 (9)	C12—C11—H11	120.0
C5—C4—C3	120.16 (9)	N5—C12—C11	118.53 (9)
C6—C5—C4	118.91 (10)	N5—C12—C7	122.41 (9)
C6—C5—H5	120.5	C11—C12—C7	119.06 (9)
C4—C5—H5	120.5		
			
C1—N1—N2—C2	0.18 (12)	C4—C5—C6—C7	0.96 (15)
C1—N3—N4—C3	178.11 (10)	C5—C6—C7—C8	178.09 (10)
C2—N3—N4—C3	−4.93 (17)	C5—C6—C7—C12	−1.59 (15)
N2—N1—C1—N3	−0.19 (12)	C6—C7—C8—C9	177.65 (10)
C2—N3—C1—N1	0.12 (12)	C12—C7—C8—C9	−2.68 (15)
N4—N3—C1—N1	177.84 (9)	C7—C8—C9—C10	0.74 (16)
N1—N2—C2—N3	−0.11 (13)	C8—C9—C10—C11	1.74 (16)
C1—N3—C2—N2	0.00 (13)	C9—C10—C11—C12	−2.19 (16)
N4—N3—C2—N2	−177.30 (10)	C4—N5—C12—C11	178.64 (9)
N3—N4—C3—C4	178.88 (8)	C4—N5—C12—C7	−1.26 (14)
C12—N5—C4—C5	0.58 (15)	C10—C11—C12—N5	−179.70 (9)
C12—N5—C4—C3	−179.61 (8)	C10—C11—C12—C7	0.21 (15)
N4—C3—C4—N5	172.73 (9)	C8—C7—C12—N5	−177.90 (9)
N4—C3—C4—C5	−7.45 (15)	C6—C7—C12—N5	1.78 (15)
N5—C4—C5—C6	−0.45 (16)	C8—C7—C12—C11	2.20 (15)
C3—C4—C5—C6	179.75 (9)	C6—C7—C12—C11	−178.12 (9)

### Hydrogen-bond geometry (Å, º)

**Table:**

*D*—H···*A*	*D*—H	H···*A*	*D*···*A*	*D*—H···*A*
O1*W*—H1*W*···N1	0.914 (18)	1.939 (18)	2.8422 (11)	169.3 (17)
